# Genetic evidence on the causality between gut microbiota and various asthma phenotypes: a two-sample Mendelian randomization study

**DOI:** 10.3389/fcimb.2023.1270067

**Published:** 2024-01-11

**Authors:** Zi-Xuan Cheng, Yi-Xing Wu, Zhi-Jun Jie, Xing-Jing Li, Jing Zhang

**Affiliations:** ^1^ Department of Pulmonary and Critical Care Medicine, Zhongshan Hospital, Shanghai Medical College, Fudan University, Shanghai, China; ^2^ Department of Respiratory and Critical Care Medicine, the Fifth People’s Hospital of Shanghai, Fudan University, Shanghai, China; ^3^ Department of Respiratory Medicine, Zhongshan Hospital Wusong Branch, Fudan University, Shanghai, China

**Keywords:** asthma, gut-lung axis, gut microbiota, Mendelian randomization analysis, phenotypes, endotypes

## Abstract

**Introduction:**

Asthma is a multifarious disease that manifests in various phenotypes. Among the various factors that contribute to the development of asthma, the gut microbiota has recently emerged as a compelling area of investigation. This study aims to investigate the causal relationships between gut microbiota and distinct asthma phenotypes.

**Methods:**

The genome-wide association study (GWAS) summary statistics for 211 gut microbial taxa were used as study exposure. Five traits pertaining to various asthma phenotypes (asthma, allergic asthma, childhood asthma, suggestive for eosinophilic asthma and obesity-related asthma) were included as study outcome. We conducted Mendelian randomization (MR) analysis and sensitivity analysis for each bacterial taxa and asthma phenotypes.

**Result:**

We discovered a total of 58 associations that exhibited evidence of causality. Out of these, 4 associations remained significant even after applying multiple correction. An increased risk of asthma was causally associated with higher abundance of genus *Holdemanella* (OR = 1.11; CI: 1.05-1.17; p = 0.027), genus *Oxalobacter* (OR = 1.09; CI: 1.04-1.15; p = 0.025) and genus *Butyricimonas* (OR = 1.14; CI: 1.06-1.22; p = 0.027). Order *NB1n* was causally linked with an increased risk of obesity-related asthma (OR = 1.17; CI: 1.07-1.29; p = 0.015). There was limited overlap among the taxa that exhibited potential causal relationships with distinct asthma phenotypes.

**Conclusion:**

Our research has provided genetic evidence that establishes multiple causal relationships between the gut microbiota and distinct asthma phenotypes, supporting the role of the gut microbiota in various asthma phenotypes. It is possible that different taxa play a role in the development of distinct asthma phenotypes. The causal relationships identified in this study require further investigation.

## 1 Introduction

Asthma, a chronic respiratory disease, manifests a spectrum of symptoms ranging from wheezing, shortness of breath, cough and chest tightness, all accompanied by variable airflow obstruction. It is a multifarious disease, characterized by a myriad of endotypes and phenotypes. In brief, asthma manifests predominantly in two major endotypes: T-helper cell type 2 (Th2)-high (eosinophilic) and Th2-low (non-eosinophilic), also known as type 2 (T2) asthma and non-T2 asthma, respectively. Th2-high phenotypes are delineated into three distinct subgroups: early-onset allergic asthma, late-onset eosinophilic asthma, and aspirin-exacerbated respiratory disease. Conversely, Th2-low phenotypes are classified based on clinical attributes encompassing obesity, smoking and age (Kuruvilla et al., 2019).

The multifarious nature of asthma yields varying responses to therapy, resulting in severe cases that persist despite potent treatments. Severe asthma is observed in up to 10% of adults and 2.5% of children diagnosed with asthma, bearing a greater burden of asthma-related morbidity and mortality (Kuruvilla et al., 2019; Brusselle and Koppelman, 2022). Therefore, there is a necessity for an in-depth study of the intricate mechanisms underlying the diverse asthma endotypes and phenotypes.

Among the multitude factors that contribute to the development of asthma, the gut microbiota, a pivotal agent in immune system development, has recently emerged as a compelling area of investigation. Recent discoveries indicate that alterations in gut microbial composition and function are closely linked to fluctuations in immune responses and the occurrence of respiratory diseases, including asthma (Budden et al., 2017). Disruptions in the gut microbiota during early life have been associated with an increased susceptibility to asthma (Fujimura et al., 2016). Although the overall composition of the gut microbial community tends to remain relatively stable in both children and adults, specific differences in certain taxa are believed to play a potential role in the development of asthma (Budden et al., 2017; Barcik et al., 2020).

Microecological studies typically involve collecting fecal samples from patients and analyzing them through sequencing techniques like 16S rRNA gene sequencing. These studies aim to compare the alterations in the microbial composition between individuals with a specific disease and healthy individuals. However, conflicting results are often observed in similar studies due to the absence of standardized sampling methods and longitudinal study designs (Knight et al., 2018; Combrink et al., 2023). Moreover, these observational studies are often hindered by confounding factors (e.g., antibiotic or corticosteroid use) and reverse causality. Thus, establishing a causal relationship between gut microbiota and asthma is challenging.

To overcome these obstacles, we employed a two-sample Mendelian randomization (MR) study, an effective analytical tool that utilizes genetic variants as instrumental variables (IVs) to infer causality between potentially modifiable risk factors and diseases. MR uses genetic variation to mimic randomized controlled trials (RCTs). Suppose there is a single nucleotide polymorphism (SNP) that is known to influence a certain phenotype (exposure). By following Mendel’s laws of inheritance and the fixed nature of germline genotypes, the alleles received by an individual at this SNP are expected to be random. In this “natural experiment,” the SNP is considered an IV, and observing an individual’s genotype at this SNP is similar to randomly assigning them to a treatment or control group in an RCT (Hemani et al., 2018). MR analysis evaluate the causal relationship between the exposure and outcome by comparing the impact of SNPs on the outcome to their impact on the exposure (Davies et al., 2018). SNPs are treated as independent RCTs, and meta-analysis tools can be adapted to combine the results from each SNP, providing a more powerful overall causal estimate. Two-sample MR utilizes data from separate GWAS studies for the exposure and the outcome. The independent transmission of alleles from parents to offspring ensures that confounding factors do not affect the study of health outcomes. Additionally, germline genotypes remain unaffected by disease processes, preventing reverse causality from impacting associations between genotype and disease outcomes (Lawlor et al., 2008).

In previous MR studies, Shi and colleagues have revealed the causal interplay between gut microbiota and various chronic respiratory diseases, including unspecified asthma (Shi et al., 2023). As the scientific spotlight on asthma moves towards understanding the etiologic molecular mechanisms, our objective was to further investigate the causal relationships between gut microbiota and distinct asthma phenotypes. This study aimed to strengthen the basis for precision gut-microbiota-mediated therapeutic strategies, thereby adding genetic evidence to the evolving landscape of asthma management.

## 2 Materials and methods

### 2.1 Study design

**Figure 1:**
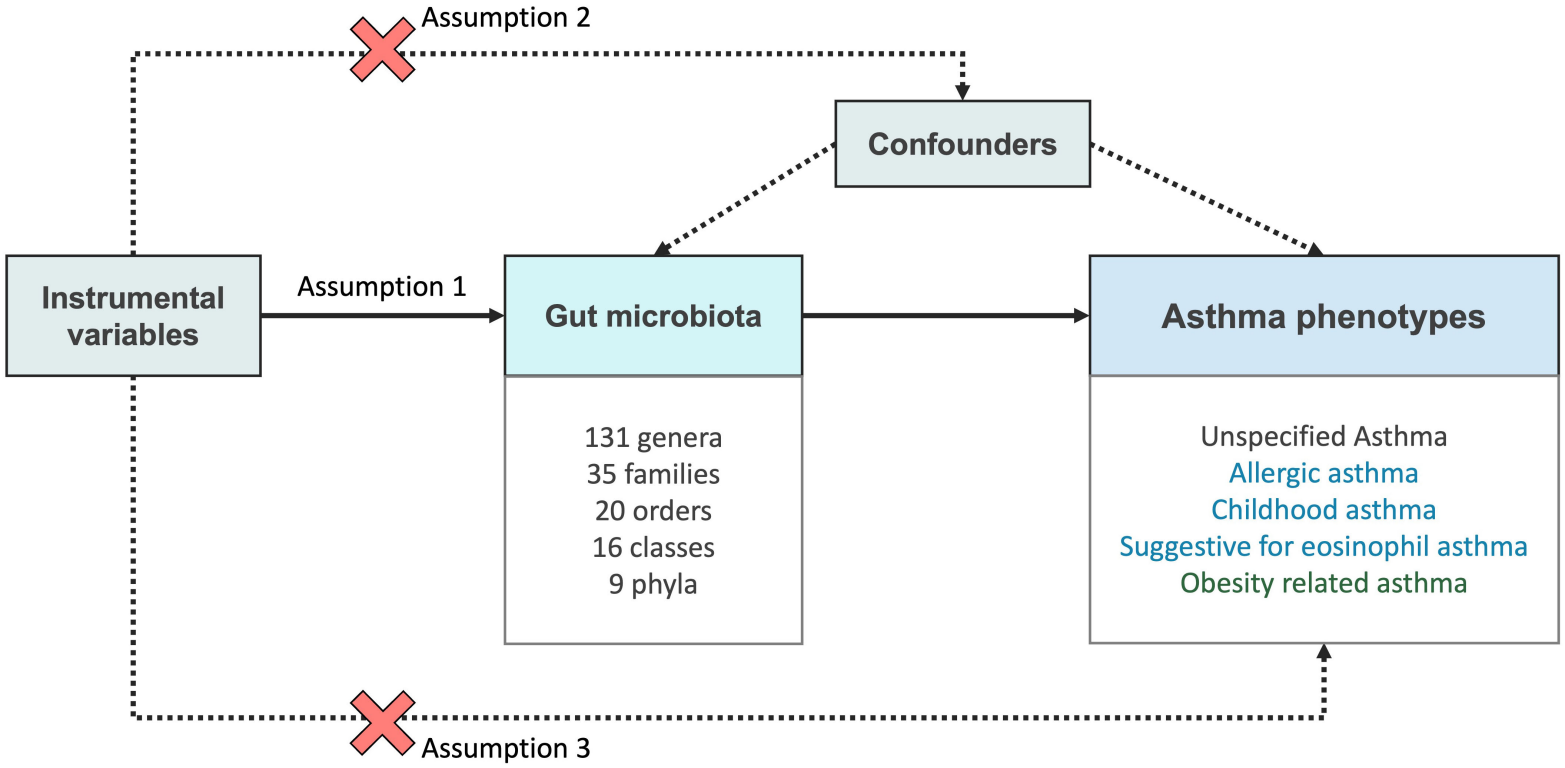
Overview of the MR framework used to investigate the causal effect of gut microbiota and asthma phenotypes. In Mendelian randomization (MR), three core assumptions are used to test for causality between the exposure (gut microbiota) and the outcome (asthma phenotypes). Assumption 1 (Relevance assumption): The genetic variants used as instrumental variables (IVs) are related to the exposure of interest. Assumption 2 (Independence assumption): The IVs are not related to any other factors (confounders) that may influence both the exposure and the outcome, apart from its effect on the exposure. Assumption 3 (Exclusion restriction): The genetic variant affects the outcome only through its impact on the exposure and not through any alternative pathways.

The aim of this MR analysis was to examine the proposition that the gut microbial taxa exert causal impacts on asthma phenotypes, and to offer quantifiable evaluations for each relationship between exposure and outcome. A comprehensive set of 211 microbial traits (including 9 phyla, 16 classes, 20 orders, 35 families, and 131 genera) were incorporated as exposure. Regarding the study outcomes, we have included summary statistics pertaining to various asthma phenotypes, including unspecified asthma, allergic asthma, childhood asthma, suggestive for eosinophilic asthma, and obesity-related asthma. While allergic asthma, childhood asthma, and suggestive for eosinophilic asthma adopt the T2 asthma classification, obesity-related asthma adopts the classification of non-T2 asthma. **Figure 1** illustrates three key assumptions in MR analysis. The first assumption states that IVs are strongly associated with the exposure of interest. To address this assumption, we incorporated SNPs with genome-wide significance with a p value <1 × 10^-5^ and excluding SNPs with a F-statistic below 10. The second assumption asserts that IVs have no association with any confounding factors that may influence relationship between exposure and outcome, this is satisfied due to the random distribution of alleles during gamete formation. Lastly, the third assumption posits that the effect of the IVs on the outcome are solely mediated through their impact on the exposure. To fulfill this assumption, we conducted sensitivity analysis to examine for pleiotropy. Any relationships that were identified to have pleiotropic effects were excluded from our results. This study was conducted in strict adherence to the STROBE-MR guideline (Lawlor et al., 2008).

### 2.2 Study exposures and outcomes

The IVs, which are human SNPs associated with the abundance of gut microbiota, were procured from a far-reaching Genome-Wide Association Study (GWAS) conducted by the MiBioGen consortium. This study scrutinized host genetic variations across 18,340 participants from 24 cohorts, with a predominance of European lineage accounting for 85% (Shi et al., 2023) (Kurilshikov et al., 2021). (**Supplementary Table S8**) A total of 211 taxa (9 phyla, 16 classes, 20 orders, 35 families, and 131 genera) were included, as discerned through 16S rRNA gene sequencing techniques. Asthma phenotypic GWAS summary statistics were extracted from the eighth analytical round of the FinnGen biobank 8 (accessed on the 29^th^ of April, 2023) (Kurilshikov et al., 2021). We selectively culled a quintet of conditions for further analysis, including asthma (unspecified), allergic asthma, childhood asthma, suggestive for eosinophilic asthma and obesity-related asthma. There was minimal overlap between the individuals included in the samples of exposure and outcome. Relevant information regarding the GWAS summary statistics is presented in **Supplementary Table S8** for reference.

### 2.3 Selection of instrumental variables and data harmonization

SNPs with genome-wide significance, defined as having a p value <1 × 10^-5^ were considered to be significantly associated with the gut microbiota. (**Supplementary Table S9**) These SNPs were subsequently included to perform further analyses. These variants were clumped to ensure that the IVs used were independent with a clumping window of 10,000 kb and a pairwise linkage disequilibrium (LD) threshold of r^2^ < 0.001. To avoid weak instrument bias and ensure the strength of the IVs, we calculated the F-statistic. Any SNPs with an F-statistic less than 10 were excluded from the analysis. Variant harmonization was performed by aligning the betas of different studies on the same effect allele with the TwoSampleMR package in R. Given the variability in genotyping platforms used in GWAS, it is possible that specific SNPs associated with the gut microbiota may not be present in the outcome dataset. These missing SNPs were excluded in this study.

### 2.4 Primary Mendelian randomization analyses

The primary method employed for causal inference was the inverse variance weighted (IVW) method. This method combines the ratio estimates derived from each genetic instrument in a meta-analysis model. The IVW method assigns greater weight to the ratio estimates with lower variance, thereby providing a more reliable estimate of the causal relationship between the exposure and the outcome variables (Gagnon et al., 2023). The plausibility of the results obtained from the IVW method relies on each SNP satisfying the assumptions of MR. Relationships that passed a nominal p-value significance threshold of 0.05 according to IVW analysis are considered possible causal relationships. Relationships that met the nominal p-value significance threshold of 0.05 as determined by the IVW method were considered as possible causal relationships.

In order to further assess the robustness of the findings, we employed supplementary analysis methods, including MR-Egger, weighted median, simple mode, weighted mode and MR-PRESSO. MR-Egger is utilized to detect violations of the MR assumptions, such as horizontal pleiotropy, and provides an effect estimate that is not subjected to these violations (Kurki, 2023). The weighted median method combines the ratio estimates from the genetic instruments using a median-based approach, which can provide reliable estimates even if up to 50% of the instruments are invalid. The simple mode and weighted mode methods consider the majority or weighted majority of the genetic instrument estimates, respectively, to ascertain the direction and strength of the causal relationship (Gagnon et al., 2023). MR-PRESSO is a general test for the presence of outliers, was used to identify and remove genetic variants that significantly contributed to heterogeneity through a simulation approach (Bowden et al., 2015). By incorporating these supplementary analysis methods, we can evaluate the consistency of the results and gain a more comprehensive understanding of the causal associations while considering potential violations of the MR assumptions.

For associations with IVW-MR p-value <0.05, we applied a Benjamini Hochberg (BH) correction for multiple testing to reduce the propensity of false positive finding. The relationships that remained significant after BH correction were considered as significant causal relationships. In addition, we performed reverse analysis to examine the reverse causal association. All the statistical analyses were performed using R version 4.2.3.

### 2.5 Sensitivity analyses

To evaluate the robustness of our primary causal estimate for associations with IVW-MR p-value <0.05, we conducted sensitivity analyses. Both IVW and MR-Egger were applied to assess the heterogeneity of effects using Cochran’s Q statistic. To evaluate the presence of horizontal pleiotropy, we employed the MR-Egger intercept and MR-PRESSO Global test. If any results indicated the presence of pleiotropy with a significance level of p <0.05, those results were excluded (Bowden et al., 2015; Kurki, 2023). Furthermore, we conducted a leave-one-out analysis to examine the outliers and the stability of the results. These sensitivity analyses allowed us to ensure the reliability of our findings.

## 3 Result

The objective of this study was to investigate the causal effects of specific gut microbial taxa on asthma phenotypes. (**Figure 1**) A comprehensive set of 211 microbial traits were incorporated as exposure. As for the study outcomes, we included summary statistics related to various asthma phenotypes. These phenotypes comprised unspecified asthma, allergic asthma, childhood asthma, eosinophilic asthma, and obesity-related asthma. The triad of allergic asthma, childhood asthma, and suggestive for eosinophilic asthma adopt the T2 asthma classification, whereas obesity-related asthma adopts the non-T2 asthma classification.

### 3.1 Selection of instrumental variables and data harmonization

**Figure 2:**
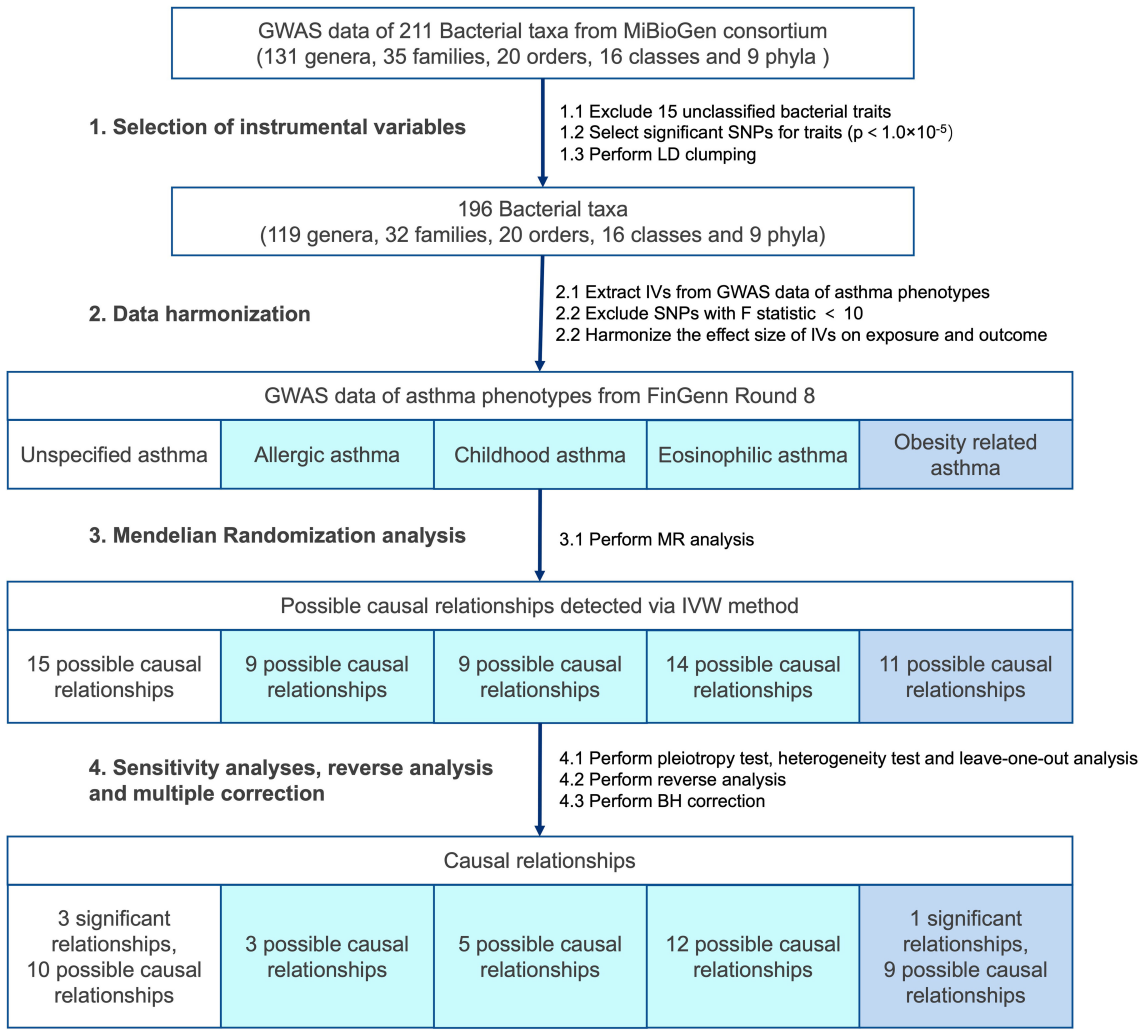
Overall workflow of the study. GWAS, Genome-Wide Association Study; SNP, single nucleotide polymorphisms; LD, linkage disequilibrium; IVs, instrumental variables; BH, Benjamini-Hochberg.

**Figure 2** depicts the overall workflow of the study. The full GWAS summary statistics related to the gut microbiota were obtained from MiBioGen Consortium, which involved the coordination of 16S rRNA gene sequencing profiles and human genotyping data from a total of 18,340 participants. The study encompassed 211 different gut bacterial taxa (Kurilshikov et al., 2021). Among the 211 bacterial taxa, 15 unknown taxa that could not be classified by 16S rRNA sequencing were excluded, leaving 196 taxa for subsequent MR analysis. The human SNPs that were associated with the 196 taxa were selected for further analysis. We selected only exposures that had at least three independent genetic instruments with a minimum significance level of p-value < 1 × 10^-5^. In MR analysis, it is crucial to ensure the independence of the variables chosen. The presence of strong LD among the included IVs can lead to biased results (Hemani et al., 2018). Thus, the SNPs were then clumped by LD to ensure the independence of the IVs. In order to avoid weak instrumental bias, we excluded SNPs with mean F statistics < 10. This step helps to ensure the reliability and robustness of our IVs in the study. We proceeded to harmonize the effect sizes of these variants on the exposure and the outcome for consistency and comparability.

### 3.2 Effect of gut microbial abundance on asthma phenotypes

**Figure 3:**
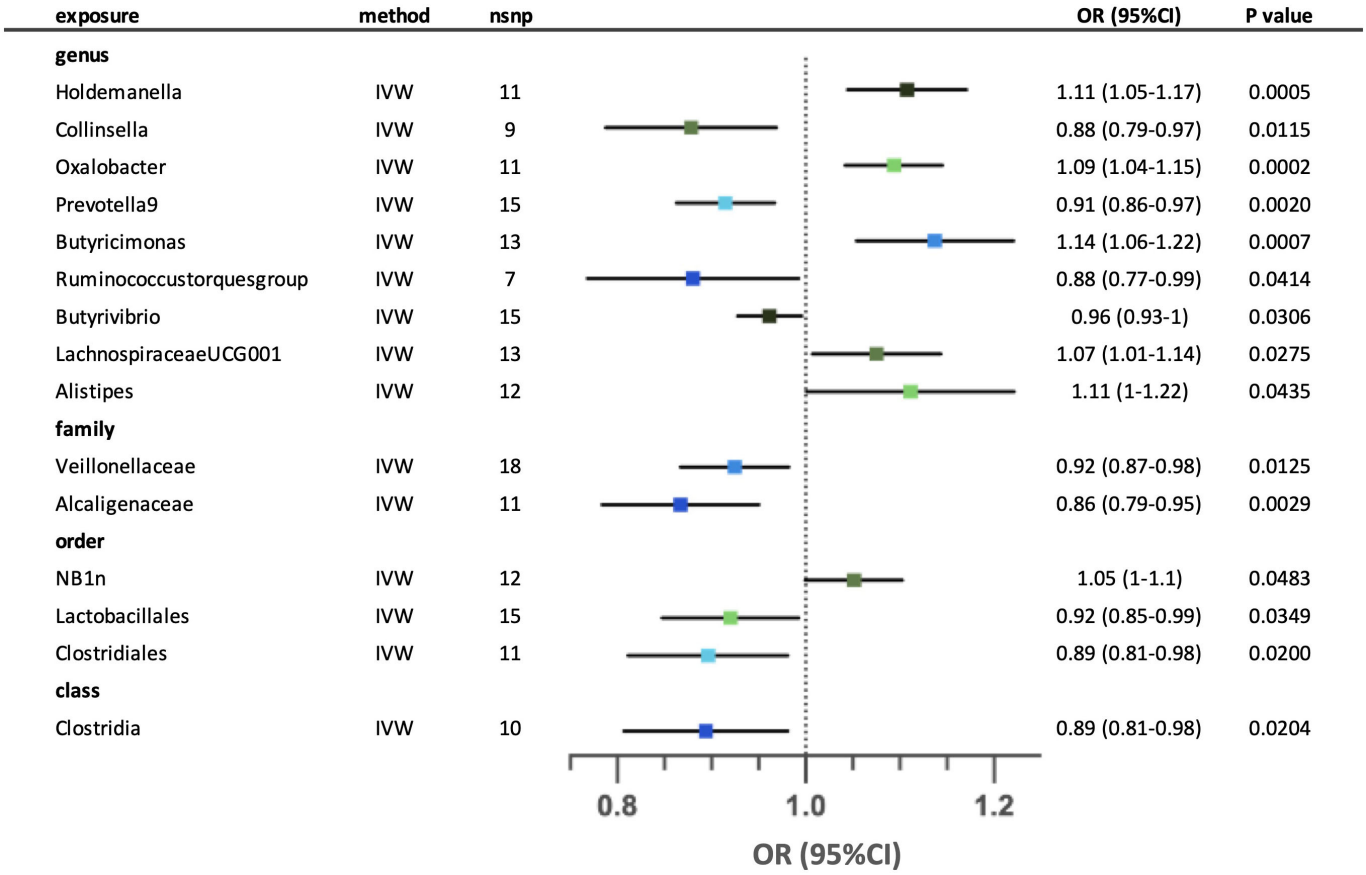
Forest plot for the possible causal relationships between gut microbiota and the risk of unspecified asthma. Fifteen possible causal relationships were identified by the inversed variance weighted method (IVW) (p<0.05). Dots depicts the point estimate of odds ratio (OR), while horizontal bars depict 95% confidence interval (CI). The number of SNPs associated with the specific taxa and included in the analysis was indicated in the “nsnp” column.

We identified fifteen possible causal relationships between the gut microbiota and the risk of unspecified asthma in the IVW analysis. (**Figure 3**) Ten out of fifteen possible causal relationships remained after sensitivity analysis. At the genus level, higher genetically predicted abundance of *Holdemanella*, *Oxalobacter*, *Butyricimonas*, *Butyrivibrio*, *LachnospiraceaeUCG001* and *Alistipes* were associated with a higher risk of asthma, whilst *Collinsella*, *Prevotella9* and *Ruminococcustorquesgroup* were associated with a lower risk of asthma. At the family level, both *Veillonellaceae* and *Alcaligenaceae* were associated with lower risk of asthma. The order NB1n and Clostridiales were correlated with higher risk of asthma, in contrast with Lactobacillales. Lastly, class Clostridia was associated with lower risk of asthma. The MR-Egger and MR-PRESSO tests showed that there is no horizontal pleiotropy (p >0.05). (**Supplementary Tables: S3-4**) According to the Cochrane’s Q test, negligible heterogeneity was shown in three taxa (genus *Holdemanella*, genus *Ruminococcustorquesgroup* and order Lactobacillales). (**Supplementary Table S5**) The leave-one-out analysis revealed that some SNPs might dominate the positive results in three taxa (genus *Butyrivibrio*, order NB1n and order Clostridiales). The relationships of five possible taxa (genus *Collinsella*, genus *Ruminococcustorquesgroup*, genus *Alistipes*, order Lactobacillales and class Clostridia) were excluded because the leave-one-out analysis yielded inconsistent results. (**Supplementary Table S6**) Reverse analysis demonstrates a potential association between asthma and an increased abundance of the family *Veillonellaceae*. (**Supplementary Table S7**) In our reverse analysis, we observed a potential association between asthma and an increased abundance of the family *Veillonellaceae*.

**Figure 4:**
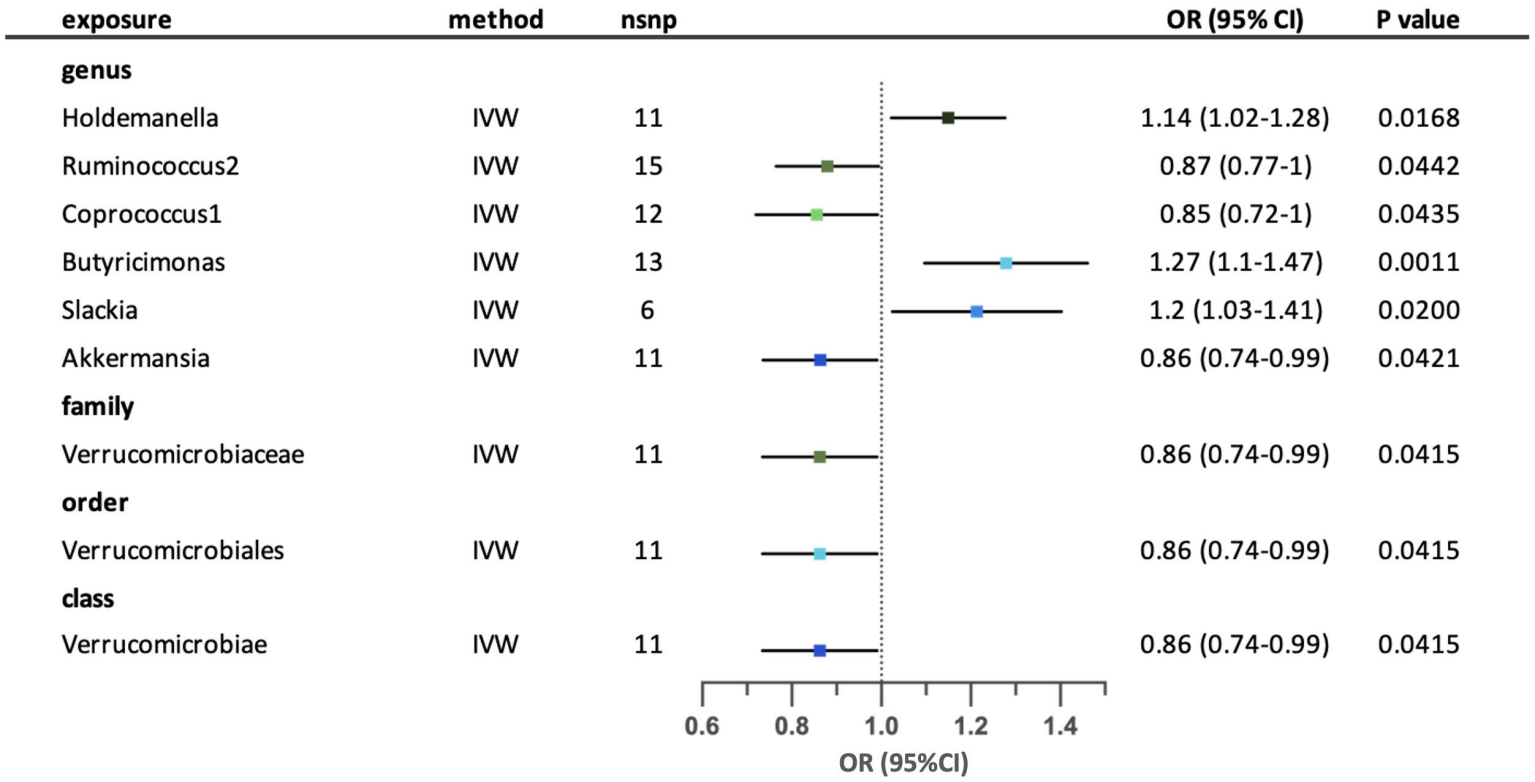
Forest plot for the possible causal relationships between gut microbiota and the risk of allergic asthma. Nine possible causal relationships were identified by the inversed variance weighted method (IVW) (p<0.05). Dots depicts the point estimate of odds ratio (OR), while horizontal bars depict 95% confidence interval (CI). The number of SNPs associated with the specific taxa and included in the analysis was indicated in the “nsnp” column.

A total of nine possible causal relationships were Identified between the gut microbiota and the risk of developing allergic asthma. (**Figure 4**) Three possible causal relationships remained after sensitivity analysis. Genus *Holdemanella*, *Butyricimonas* and *Slackia* were correlated with higher risk of allergic asthma, while *Ruminococcus2*, *Coprococcus1* and *Akkermansia* were associated with lower risk of allergic asthma. Family *Verrucomicrobiaceae*, order Verrucomicrobiale*s* and class Verrucomicrobiae have similar effects on allergic asthma by relating to lower risk of the disease. The causal links of five possible taxa (genus Coprococcus1, genus *Akkermansia*, family *Verrucomicrobiaceae*, order Verrucomicrobiales and class Verrucomicrobiae) were excluded because the existence of horizontal pleiotropy according to MR-Egger intercept or MR-PRESSO analyses. (**Supplementary Tables S3-4**) Results from Cochrane’s. Q test showed no significant heterogeneity in the remaining relationships. (**Supplementary Table S5**) The leave-one-out analysis revealed that a few particular SNPs may have overlooked the positive effects of genus *Holdemanella*. The relationship between genus *Slackia* and allergic asthma was excluded because of contradictory result according to the leave-one-out analysis. (**Supplementary Table S6**).

**Figure 5:**
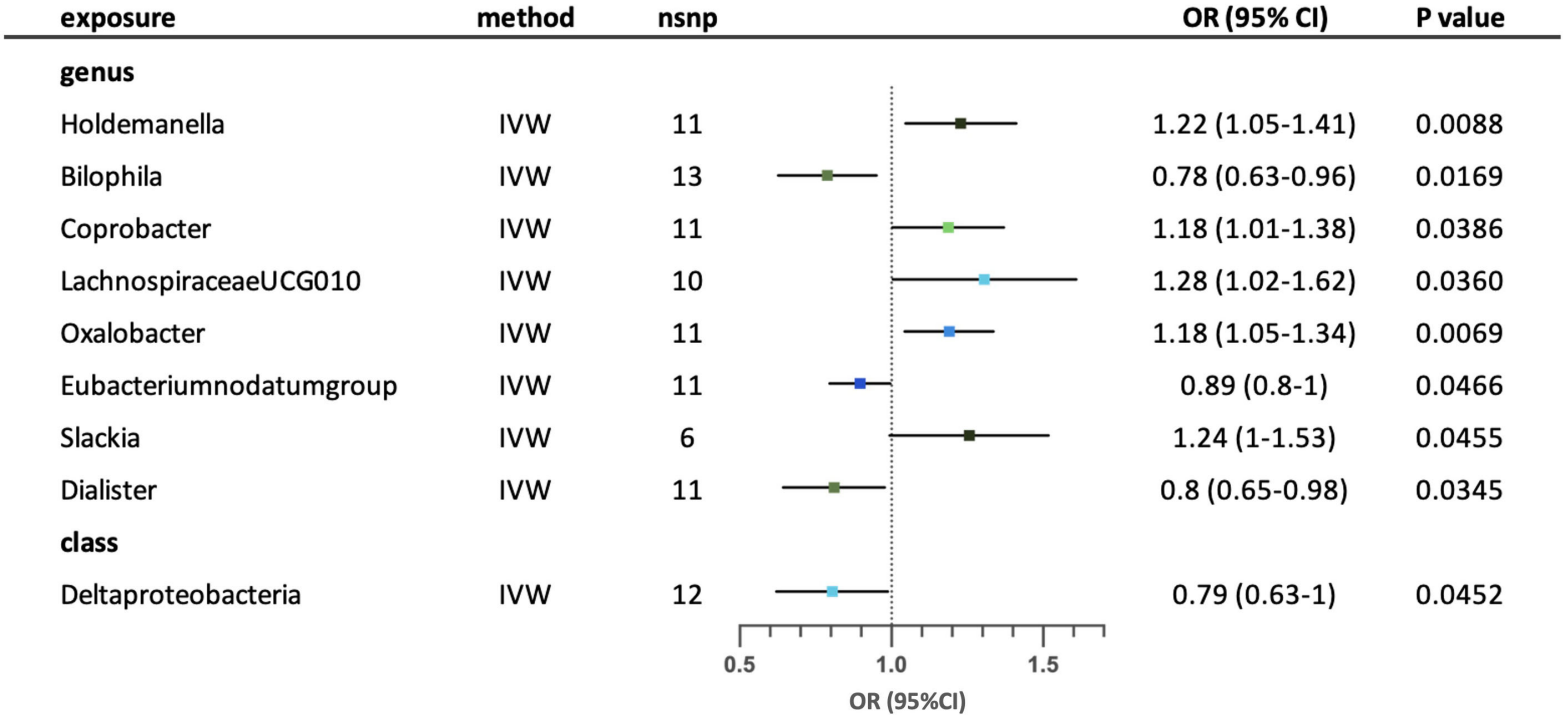
Forest plot for the possible causal relationships between gut microbiota and the risk of childhood asthma. Nine possible causal relationships were identified by the inversed variance weighted method (IVW) (p<0.05). Dots depicts the point estimate of odds ratio (OR), while horizontal bars depict 95% confidence interval (CI). The number of SNPs associated with the specific taxa and included in the analysis was indicated in the “nsnp” column.

IVW analysis revealed nine possible causal relationships between the gut microbiota and the risk of childhood asthma. (**Figure 5**) After conducting sensitivity analysis, five relationships remained. Genus *Holdemanella*, *Coprobacter*, *LachnospiraceaeUCG010*, *Oxalobacter* and *Slackia* were associated with increased risk of childhood asthma. Conversely, genus *Bilophila*, *Eubacteriumnodatumgroup* and *Dialister* were associated with reduced risk of childhood asthma. Class Deltaproteobacteria was correlated with reduced risk of childhood asthma. No significant heterogeneity and horizontal pleiotropy (p >0.05) were found according to MR-Egger intercept, MR-PRESSO and Cochrane’s Q tests. (**Supplementary Tables S3-5**) The leave-one-out analysis suggested that a few SNPs may have overlooked the positive effects of three possible taxa (genus *Bilophila*, genus *Eubacteriumnodatumgroup* and genus *Dialister*). Four possible taxa (genus *Coprobacter*, genus *LachnospiraceaeUCG010*, genus *Slackia* and class Deltaproteobacteria) were excluded due to contradictory results in leave-one-out analysis. (**Supplementary Table S6**).

**Figure 6:**
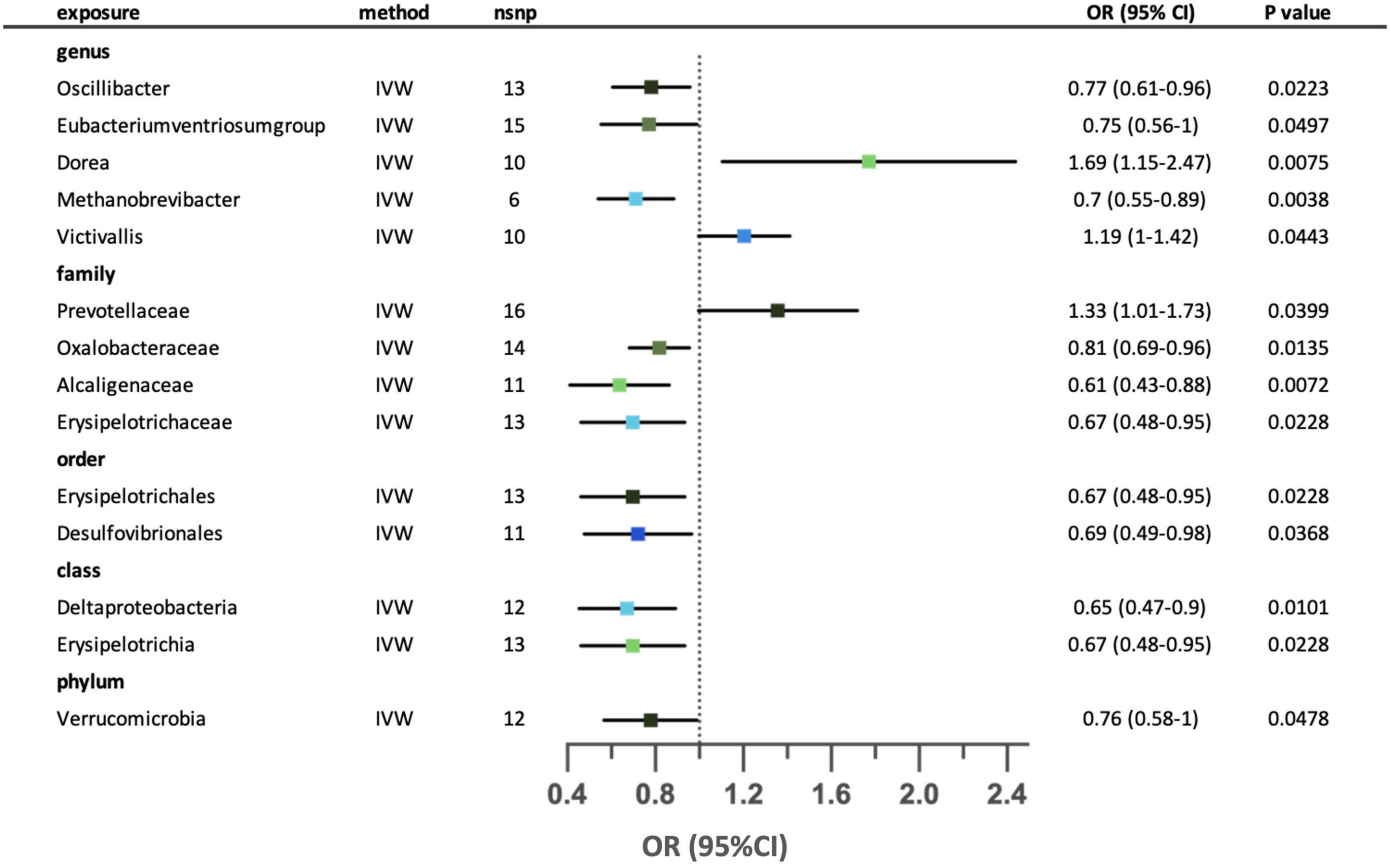
Forest plot for the possible causal relationships between gut microbiota and the risk of suggestive for eosinophilic asthma. Fourteen possible causal relationships were identified by the inversed variance weighted method (IVW) (p<0.05). Dots depicts the point estimate of odds ratio (OR), while horizontal bars depict 95% confidence interval (CI). The number of SNPs associated with the specific taxa and included in the analysis was indicated in the “nsnp” column.

Genetically predicted fourteen microbial taxa that were causally associated with an increased risk of eosinophilic asthma. (**Figure 6**) Two out of fourteen relationships were excluded after conducting sensitivity analysis. Higher genetically predicted abundance of genus *Dorea*, genus *Victivallis* and family *Prevotellaceae* were associated with increased risk of eosinophilic asthma. While higher abundance of genus *Oscillibacter*, *Eubacteriumventriosumgroup*, *Methanobrevibacter*, family *Oxalobacteraceae*, *Alcaligenaceae*, *Erysipelotrichaceae*, order Erysipelotrichales, Desulfovibrionales, class Deltaproteobacteria, Erysipelotrichia and phylum Verrucomicrobia were associated with decreased risk of eosinophilic asthma. Since horizontal pleiotropy was detected according to the MR-Egger intercept, the possible causal relationship between family *Prevotellaceae* and eosinophilic asthma was excluded. Neither horizontal pleiotropy nor heterogeneity were seen in the remaining relationships. (**Supplementary Tables S3-5**) Leave-one-out analysis revealed that the possible causal effect of three taxa (genus *Eubacteriumventriosumgroup*, genus *Victivallis* and family *Prevotellaceae)* might be dominated by part of the SNPs. The relationship between genus *Oscillibacter* and eosinophilic asthma was excluded due to contradictory result in leave-one-out analysis. (**Supplementary Table S6**).

**Figure 7:**
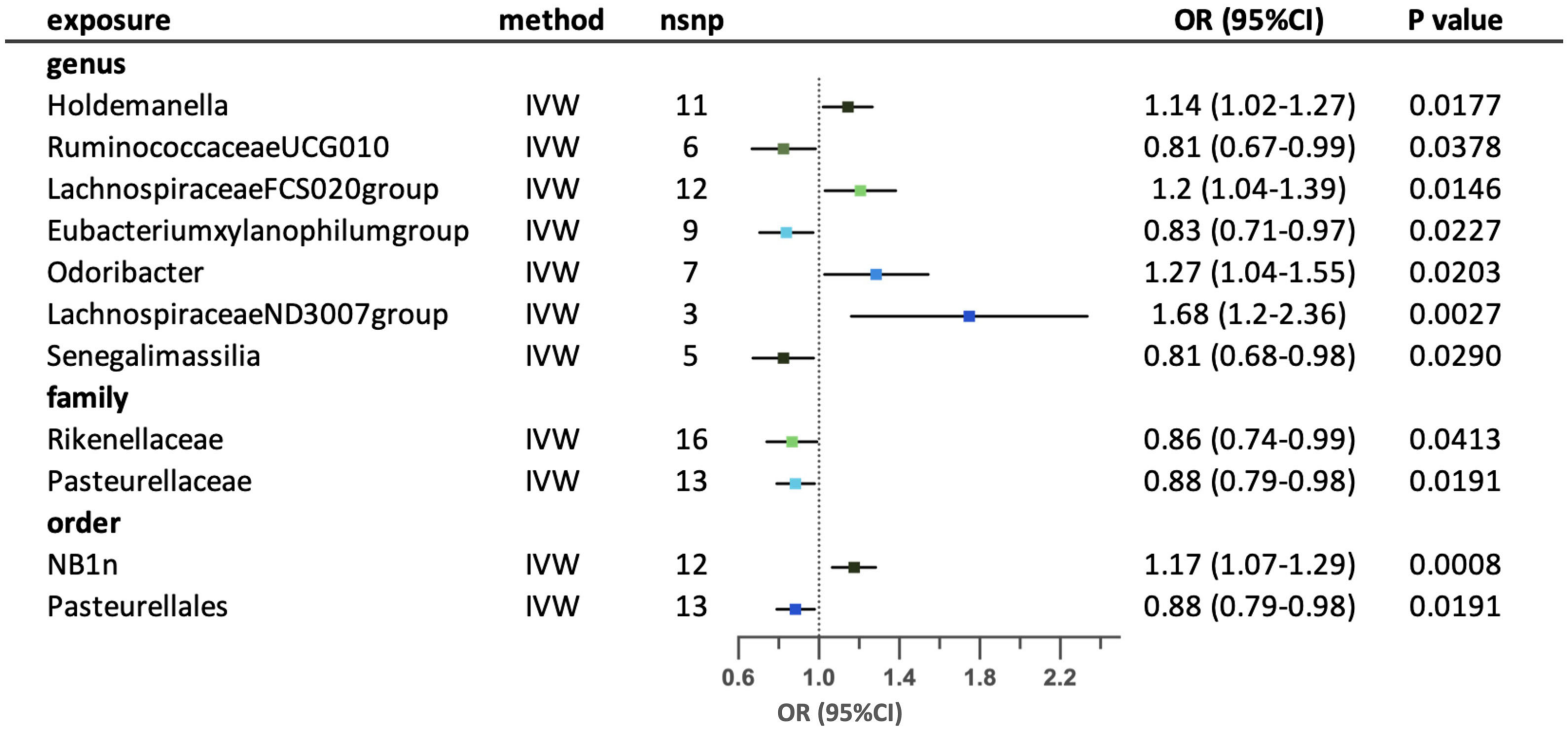
Forest plot for the possible causal relationships between gut microbiota and the risk of suggestive for obesity-related asthma. Eleven possible causal relationships were identified by the inversed variance weighted method (IVW) (p<0.05). Dots depicts the point estimate of odds ratio (OR), while horizontal bars depict 95% confidence interval (CI). The number of SNPs associated with the specific taxa and included in the analysis was indicated in the “nsnp” column.

Eleven possible causal relationships were found between the gut microbiota and obesity-related asthma. (**Figure 7**) Nine relationships remained after conducting sensitivity-analysis. At the genus level, higher genetically predicted abundance of genus *Holdemanella*, *LachnospiraceaeFCS020group*, *Eubacteriumxylanophilumgroup*, *Odoribacter* and *LachnospiraceaeND3007group* were associated with a higher risk of obesity-related asthma, in contrast with *RuminococcaceaeUCG010* and *Senegalimassilia*. Family *Rikenellaceae* and *Pasteurellaceae* were correlated with lower risk of obesity-related asthma. Order NB1n had a positive effect on the risk of obesity-related asthma, while order Pasteurellales had a negative effect on the risk of the disease. The results of MR-Egger and MR-PRESSO tests confirmed that there is no horizontal pleiotropy and the outcomes from Cochrane’s Q test demonstrated that there is no obvious heterogeneity among the selected SNPs. (**Supplementary Tables S3-5**) The leave-one-out analysis revealed that some SNPs may have overlooked the possible causal effects of three taxa (family *Rikenellaceae*, family *Pasteurellaceae* and order Pasteurellales). Two possible taxa (genus *RuminococcaceaeUCG010* and *LachnospiraceaeND3007group*) were excluded due to conflictive results in leave-one-out analysis. (**Supplementary Table S6**).

**Figure 8:**
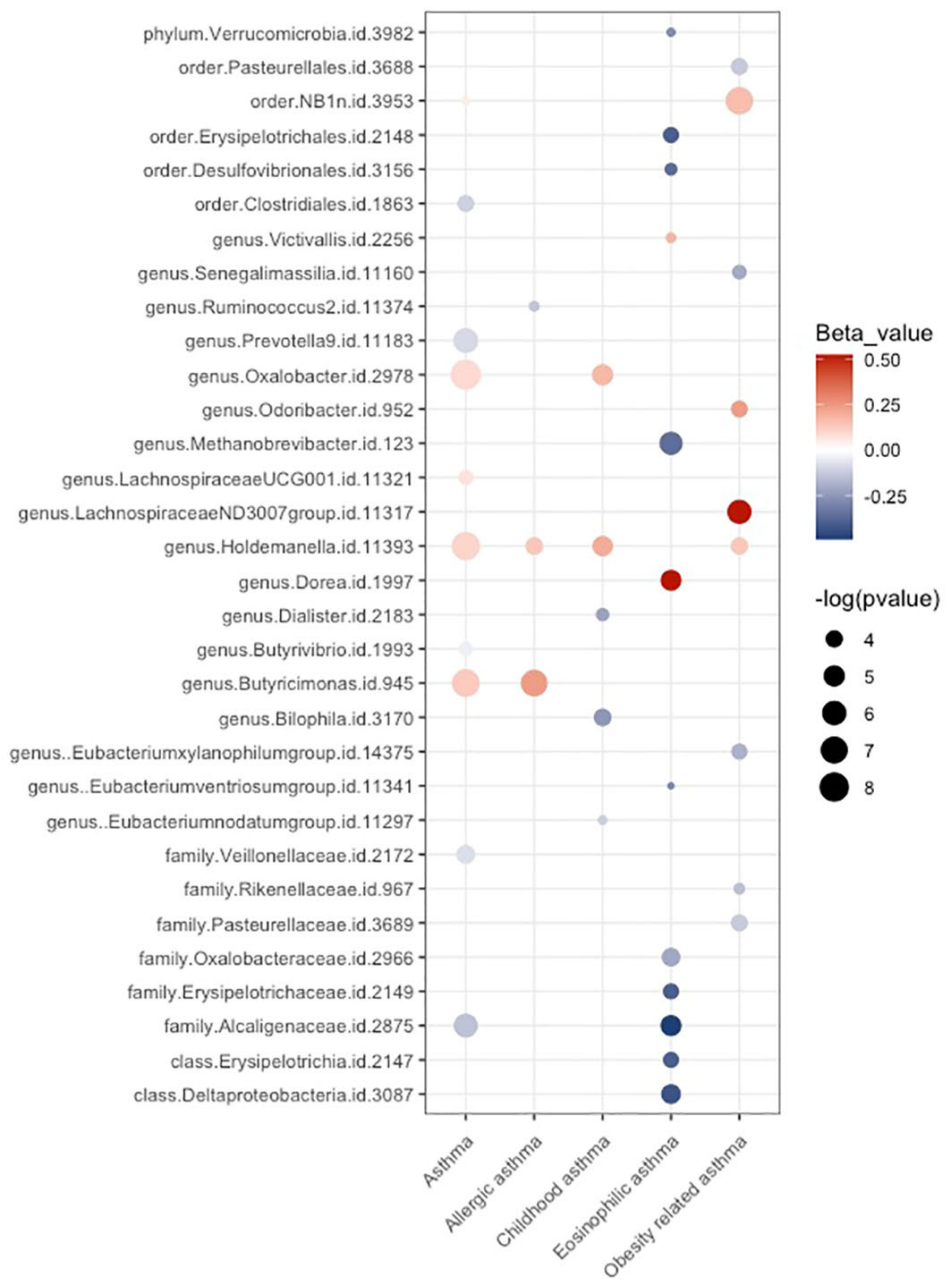
Balloon plot for the possible causal relationships between gut microbiota and the risk of asthma phenotypes after conducting sensitivity analysis. Associations with a p-value < 0.05 are represented by dots. The size of the dots corresponds the effect size. The color of dots indicates the direction of the effect. Red dots signify a positive causal effect on the outcome, while blue dots indicate a negative causal effect.

As depicted in **Figure 8**, a total of 39 possible causal associations remained after conducting sensitivity analysis. The results of our study revealed an intriguing finding: there was limited overlap among the taxa that exhibited potential causal relationships with distinct asthma phenotypes. Among the taxa examined in our study, genus *Holdemanella* emerged as having potential causal effects on four different traits. Additionally, three other taxa demonstrated potential causal effects on unspecified asthma and one specific asthma phenotype. We observed no overlap among the taxa exhibiting possible causal effects on different asthma phenotypes, except for genus *Holdemanella*. This observation indicates that different taxa might be involved in the development of distinct asthma phenotypes.

To address the possibility of false positive findings, we applied the BH correction to our analysis. Out of the 39 initial causal associations, 4 associations remained statistically significant even after BH correction (referred as significant causal relationships). (**Figure 2**) (**Table 1**) An increased risk of asthma was associated with higher abundance of three genera, including *Holdemanella* (OR = 1.11; CI: 1.05-1.17; p-IVW = 0.00050, p-BH = 0.027), *Oxalobacter* (OR = 1.09; CI: 1.04-1.15; p-IVW = 0.00021, p-BH = 0.025) and *Butyricimonas* (OR = 1.14; CI: 1.06-1.22; p-IVW = 0.00069, p-BH = 0.027). Order NB1n was causally linked with an increased risk of Obesity-related asthma (OR = 1.17; CI: 1.07-1.29; p-IVW = 0.00077, p-BH = 0.015). Notably, while we observed significant causal relationships in unspecified asthma and obesity-related asthma, no significant causal relationship remained in T2 asthma. Since the bacterial taxa that were found to have significant causal relationships with asthma showed potential causal effects on distinct asthma phenotypes, this may be attributed to the relatively small number of cases in the three T2 asthma traits. The limited sample size might have reduced the statistical power to detect significant associations in T2 asthma.

**Table 1:** Significant causal relationships between gut microbiota and the risk of asthma phenotypes after conducting multiple correction.

Exposure	Outcome	MR analyses			Pleiotropy test		Heterogeneity test	
		p (IVW)	p (BH)	OR (95% CI)	MR-PRESSO global P	MR egger intercept	Q	Leave-one-out analysis
Genus								
*Holdemanella*	Asthma (Unspecified)	0.00050	0.027	1.11 (1.05-1.17)	0.073	0.004 (p = 0.77)	17.54 (p = 0.04)	√
*Oxalobacter*		0.00021	0.025	1.09 (1.04-1.15)	0.645	-0.007 (p = 0.70)	8.35 (p = 0.50)	√
*Butyricimonas*		0.00069	0.027	1.14 (1.06-1.22)	0.116	0.018 (p = 0.21)	16.21 (p = 0.13)	√
Order								
NB1n	Obesity related asthma	0.00077	0.015	1.17 (1.07-1.29)	0.126	0.002 (p = 0.93)	14.80 (p = 0.14)	√

## 4 Discussion

Based on our current knowledge, this study represents a pioneering effort in exploring the causal connections between gut microbiota and diverse asthma phenotypes. Considering that asthma can be classified into type 2 asthma (including early-onset allergic asthma, late-onset eosinophilic asthma, and aspirin-exacerbated respiratory disease) and non-type 2 asthma (based on factors such as obesity, smoking, and age), we searched in the FinnGen biobank for GWAS data related to these phenotypes. We included five traits of asthma phenotypes that matched these classifications, namely unspecified asthma, allergic asthma, childhood asthma, suggestive for eosinophilic asthma, and obesity-related asthma. Then, we conducted MR analysis and sensitivity analysis for 211 gut microbial taxa and 5 traits of asthma. We identified a total of 58 causal relationships that met the nominal p-value significance threshold of 0.05 in our study. After performing sensitivity analysis, 40 associations remained robust. Following the multiple correction, 4 associations remained statistically significant. These causal relationships necessitate further validation and hold potential value for future studies. The immunomodulatory properties of different microbial taxa may be responsible for the observed causal relationships in our study. For example, gut microbiota-derived metabolites, specifically short-chain fatty acids (SCFAs), have shown protective effects against asthma by reducing atopic sensitization (Skrivankova et al., 2021), decreasing sputum eosinophilia (Bowden and Holmes, 2019), and alleviating airway (Verbanck et al., 2018) and lung inflammation (Cait et al., 2018; Skrivankova et al., 2021; Zou et al., 2021).

Our findings indicated that the genetically predicted abundance of three taxa, including genus *Holdemanella*, genus *Oxalobacter*, and genus *Butyricimonas*, were causally linked to an elevated risk of unspecified asthma. Additionally, the genetically predicted abundance of order NB1n is causally associated with a higher risk of obesity-related asthma. While we observed meaningful causal relationships in unspecified asthma and obesity-related asthma, no significant causal association was found in T2 asthma. This lack of significant findings in T2 asthma may be attributed to the relatively small number of cases within the three T2 asthma traits, as the bacterial taxa displaying significant causal relationships with unspecified asthma demonstrated potential effects on distinct asthma phenotypes. The limited sample size likely reduced the statistical power required to detect significant associations in T2 asthma.

Consistent with a recent MR study (Begley et al., 2018), we found that the genetically predicted abundance of genus *Holdemanella* demonstrated potential causal effects on four asthmatic traits, including unspecified asthma, allergic asthma, childhood asthma and obesity-related asthma. Interestingly, this genus was found to have a negative correlation with propionate levels in individuals with diabetes and cognitive impairment (Jin et al., 2023). This suggests that *Holdemanella* may contribute to the development of asthma through its impact on SCFAs like propionate. However, it is worth noting that *Holdemanella biformis*, a species of the *Holdemanella* genus, has exhibited protective effects against mouse colitis (Pujo et al., 2021) and type 2 diabetes (Du et al., 2022), potentially due to the production of beneficial long-chain fatty acids (LCFAs). Thus, it is crucial to remember that 16S rRNA sequencing can only classify microbial taxa at the genus level, and different species within the same genus may have contrasting effects on health. Similar to this case, it was reported that *Butyricimonas*, by decreasing the protective SCFA butyrate, had a protective effect on cystic fibrosis (Romaní-Pérez et al., 2021). However, it was observed that the abundance of this genus increased in patients with acute myocardial infarction. In the case of *Oxalobacter*, an MR study found that the genus had a causal effect on inflammatory bowel disease (Roduit et al., 2019). However, one species from this genus, *Oxalobacter formigenes*, has been used as a probiotic to prevent calcium oxalate kidney stone disease (McLoughlin et al., 2019). A conflicting study revealed that trauma patients who died had a relatively higher abundance of *Oxalobacter formigenes (* Trompette et al., 2014 *).*

We observed a causal relationship between the order NBn1 and obesity-related asthma. Metabolic reconstruction revealed that NB1n lacks a tricarboxylic acid cycle and instead relies on anaerobic fermentation of simple sugars for substrate level phosphorylation (Zheng et al., 2022). This unique metabolic pathway sheds light on the potential mechanisms underlying the observed relationship. Furthermore, a recent MR study revealed a protective effect of NB1n against upper urinary urolithiasis (Liu et al., 2022). These contrasting findings highlight the complexity of NB1n’s interactions with human health and disease. To fully comprehend the role of this genus in maintaining health or contributing to disease development, further research is necessary.

Observational and longitudinal studies have identified biomarkers that can differentiate individuals with asthma from healthy controls. Consistently, our study revealed that some of these biomarkers, such as increased *Lachnospiraceae* (Burmeister et al., 2020), decreased Clostridia (Daniel et al., 2021) and decreased *Veillonella* (Dayama et al., 2020), have potential causal effects on asthma. In the study conducted by Begley et al., it was discovered that individuals with asthma had increased levels of *Lachnospiraceae*. Furthermore, these bacteria demonstrated an independent ability to differentiate between asthmatic and healthy individuals (Burmeister et al., 2020). Multiple studies have also documented higher abundance of *Lachnospiraceae* members in asthmatic mouse models compared to control groups, with a significant positive correlation observed between these bacteria and immunoglobulin E levels as well as the percentage of eosinophils (Skennerton et al., 2016; Han et al., 2021; Tsukuda et al., 2021; Zhang et al., 2023). Zheng et al. found that the relative abundance of members from Clostridia was lower in subjects with asthma (Daniel et al., 2021). Notably, a longitudinal study revealed that the abundance of Clostridiales had association to increased level of fecal butyrate in early life (Arrieta et al., 2015). Thus, it is highly possible that Clostridiales develops protective effects through producing beneficial butyrate and relieving airway and lung inflammation in asthma. In a study conducted by Arrieta et al., it was observed that children at risk of developing asthma exhibited a significant decrease in the relative abundance of *Veillonella*. This reduction was accompanied by a decrease in fecal acetate levels (Dayama et al., 2020), indicating a potential mechanism wherein the loss of protective fecal acetate could potentially contribute to the development of asthma. However, inconsistencies were also found when comparing our study to others. Fujimura et al. discovered that different compositions of neonatal human gut microbiota were linked to varying risks of childhood asthma. They observed that lower levels of *Bifidobacteria*, *Lactobacillus*, *Faecalibacterium*, and *Akkermansia* were associated with an increased likelihood of developing childhood asthma (Fujimura et al., 2016). In our study, we did not find any causal effects of these taxa on childhood asthma. To gain a more comprehensive understanding of the relationship between microbiota and asthma phenotypes, further studies from diverse perspectives are essential.

Our study yielded an intriguing finding: there was limited overlap among the taxa that exhibited potential causal relationships with distinct asthma phenotypes. This finding highlights the complexity of the relationship between gut microbial taxa and the development of asthma. The heterogeneity of various asthmatic phenotypes also exists in the gut microbiome. Various taxa may be involved in the development of distinct asthma phenotypes. This could involve different biological pathways, interactions with the immune system, or other factors that influence the development and progression of asthma phenotypes. Begley et al. identified three distinct groups of asthma patients based on their gut microbiota composition. Furthermore, these groups showed variations in inflammatory mediators, including sputum interleukin (IL) -8 and IL-1β (Burmeister et al., 2020). Zheng et al. discovered that allergic asthma patients and non-allergic asthma children exhibited notable differences in their gut microbiota composition and metabolites. This suggests that the unique gut microbiome and microbiome-derived metabolites may have a significant impact on the modulation of distinct asthma phenotypes (Daniel et al., 2021). Zou et al. made similar observations and demonstrated that variations in gut microbiota composition could effectively differentiate patients diagnosed with allergic asthma (IL-4 high) from non-allergic asthma (IL-4 low) patients (Hu et al., 2023). Taken together, there is a possible connection between gut microbiota composition and the presence of different types of inflammation, such as eosinophilic or neutrophilic, which could potentially contribute to the development of distinct asthma phenotypes. When targeting precision treatment for various asthmatic phenotypes, it is crucial to take into account the differences in the gut microbiome.

The development and management of various asthma phenotypes are influenced by diverse factors. In this study, we have focused on investigating microecological differences as one aspect of understanding asthma’s pathogenesis. However, it is important to note that a comprehensive consideration of multiple factors is necessary to fully comprehend the intricate nature of asthma. Furthermore, it is crucial to consider the potential impact of different treatments, such as the use of antibiotics, on the gut microbiome. These treatments can have varying effects on the composition and function of the microbiome. Therefore, when targeting the gut microbiome for therapeutic purposes, it is necessary to take into account the potential consequences. The microecological differences observed in this study provide an entry point for exploring the complex interplay between the microbiome and asthma. By identifying and addressing the unique microbiome characteristics associated with different asthma phenotypes, we can potentially develop tailored approaches to effectively manage and treat the condition. We believe that targeting specific subtypes of the microbiome shows great promise for advancing precision diagnosis and treatment of asthma.

The utilization of a MR design is a notable advantage. By using genetic variants as IVs, MR analysis provides a way to explore causal relationships between exposures and outcomes. The random allocation of genetic variants during conception helps to mitigate potential confounding factors that may be present in observational studies. Moreover, our study benefits from the inclusion of the largest publicly available GWAS datasets, ensuring a robust and comprehensive analysis. The use of large datasets increases the statistical power of the MR analysis, enhancing our ability to detect and estimate causal effects accurately. Due to the absence of standardized sampling methods and longitudinal study, microecological studies often yield contradictory results (Knight et al., 2018; Combrink et al., 2023). Moreover, confounding factors and reverse causality can often hinder the results of observational studies. Hence, we hope to contribute to a better understanding of the intricate interplay between gut microbiota composition and the development of asthma phenotypes by employing a robust genetic framework.

We must acknowledge the limitations of our study. Firstly, it is important to recognize that the microbiome, being an exposure phenotype, is influenced by numerous variants with relatively small effect sizes. The complex and diverse nature of the gut microbiome means that it is influenced by numerous variants with small effects. To address this complexity and increase statistical power, we have used a less stringent p-value threshold in line with previous studies (Wang et al., 2021). By adopting this approach, we can incorporate a larger number of IVs into our analysis, thereby facilitating the use of sensitivity analysis and bolstering statistical power. However, this approach carries the risk of including false-positive variants. In order to mitigate the influence of weak instruments, we have excluded SNPs with an F-statistic below 10. Secondly, the microbiome GWAS used in our analysis did not target the complete 16S rRNA gene, which limits our ability to identify potential therapeutic targets with sufficient taxonomic resolution. Thirdly, our sample primarily consisted of individuals of European ancestry to reduce bias from population stratification. However, it is important to acknowledge that this restriction in sample diversity somewhat limits the generalizability of our findings to individuals of different ethnic groups.

Collectively, our findings underscore the diverse and complex nature of asthma and offer compelling genetic evidence supporting a causal association between gut microbiota and distinct asthma phenotypes. The causal relationships identified in our study highlighted the role of gut microbiota in the development of various asthma phenotypes. It is possible that different taxa play a role in the development of various asthma phenotypes. These findings underscore the importance of further research and replication studies to validate and understand the specific mechanisms underlying these observed causal relationships. By unraveling the distinct contributions of bacterial taxa to different asthma phenotypes, we can gain valuable insights into the complex pathogenesis of asthma and potentially identify targeted interventions for specific subgroups of patients.

## Data availability statement

The original contributions presented in the study are included in the article/**Supplementary Material**. Further inquiries can be directed to the corresponding author.

## Ethics statement

Ethical approval was not required for the study involving humans in accordance with the local legislation and institutional requirements. Written informed consent to participate in this study was not required from the participants or the participants’ legal guardians/next of kin in accordance with the national legislation and the institutional requirements.

## Author contributions

Z-XC: Data curation, Formal Analysis, Investigation, Methodology, Visualization, Writing – original draft, Writing – review & editing. Y-XW: Writing – original draft. Z-JJ: Conceptualization, Writing – review & editing. X-JL: Conceptualization, Writing – review & editing. JZ: Conceptualization, Funding acquisition, Supervision, Validation, Writing – review & editing.
